# Metformin treatment reduces temozolomide resistance of glioblastoma cells

**DOI:** 10.18632/oncotarget.12859

**Published:** 2016-10-24

**Authors:** Seung Ho Yang, Shenglan Li, Guangrong Lu, Haipeng Xue, Dong H. Kim, Jay-Jiguang Zhu, Ying Liu

**Affiliations:** ^1^ Department of Neurosurgery, McGovern Medical School, University of Texas Health Science Center at Houston, Houston, Texas, USA; ^2^ Department of Neurosurgery, St. Vincent's Hospital, College of Medicine, The Catholic University of Korea, Suwon, South Korea; ^3^ Center for Stem Cell and Regenerative Medicine, The Brown Foundation Institute of Molecular Medicine, University of Texas Health Science Center at Houston, Houston, Texas, USA

**Keywords:** glioblastoma, metformin, temozolomide resistance, SOX2, global gene expression

## Abstract

It has been reported that metformin acts synergistically with temozolomide (TMZ) to inhibit proliferation of glioma cells including glioblastoma multiforme (GBM). However, the molecular mechanism underlying how metformin exerts its anti-cancer effects remains elusive. We used a combined experimental and bioinformatics approach to identify genes and complex regulatory/signal transduction networks that are involved in restoring TMZ sensitivity of GBM cells after metformin treatment. First, we established TMZ resistant GBM cell lines and found that the resistant cells regained TMZ sensitivity after metformin treatment. We further identified that metformin down-regulates SOX2 expression in TMZ-resistant glioma cells, reduces neurosphere formation capacity of glioblastoma cells, and inhibits GBM xenograft growth *in vivo*. Finally, the global gene expression profiling data reveals that multiple pathways are involved in metformin treatment related gene expression changes, including fatty acid metabolism and RNA binding and splicing pathways. Our work provided insight of the mechanisms on potential synergistic effects of TMZ and metformin in the treatment of glioblastoma, which will in turn yield potentially translational value for clinical applications.

## INTRODUCTION

Glioblastoma multiforme (GBM) is the most prevalent and deadly primary malignant brain tumor, with a median overall survival of 14.6 months [1]. Current treatments of GBM include maximum safe craniotomy, radiotherapy, and chemotherapy. Temozolomide (TMZ) is the main drug in chemotherapy regimens for newly diagnosed GBM. TMZ induces double-stranded DNA breaks in tumor cells and acts via interference with the DNA mismatch repair (MMR) pathway [2, 3]. Despite aggressive therapies, GBM recurrence is inevitable in most GBM patients [4]. This is partly due to the development of TMZ resistance, which is mediated by multiple possible mechanisms, including increased activity of DNA repair enzyme, O6-methylguanine DNA methyltransferase (MGMT) [5, 6], amplification or mutations of the epidermal growth factor receptor (EGFR) gene [7], mutations of tumor suppressor genes TP53 and PTEN [8, 9], and aberrant miRNA expression [10]. Although different therapeutic molecules have been developed to target these pathways and have demonstrated promising results from both *in vitro* and *in vivo* experimental models, these strategies often showed limited efficacy and/or intolerable side effects when applied to GBM patients.

Recent work has shown that metformin, an FDA approved anti-diabetic medication, could have anti-cancer effects in patients with a variety of cancer types including breast cancer, pancreatic cancer, colon cancer, and ovarian cancer [11–14]. In addition, metformin might have synergistic effects with TMZ treatment and enhance chemotherapy efficacy in GBM [15–17], which opens a new avenue to overcome TMZ resistance in glioma treatment. As an inexpensive, well-tolerated, first-line anti-diabetic oral drug, metformin has been reported to significantly reduce gluconeogenesis in the liver and increase insulin receptor sensitivity and glucose uptake in peripheral tissues. In addition, metformin also functions along the fatty acid metabolism pathway by de-repressing fatty acid oxidation.

Several potential mechanisms have been investigated attempting to explain the anti-cancer effects of metformin. Previous reports have identified metformin playing a role in activating AMP-activated protein kinase (AMPK)-mammalian target of rapamycin (mTOR) signaling pathway, which is important in regulating cancer cell survival, proliferation and apoptosis, as well as the process of epithelial-to-mesenchymal cells transition (EMT) phenotype [12–14]. As AKT phosphorylation is implicated in TMZ drug resistance [18, 19], it is possible that metformin might act via inhibition of AKT phosphorylation in cancer cells, thus inhibiting cancer proliferation, metastasis, and drug resistance [16, 20]. Metformin has also been found to reverse or reduce drug resistance through inhibition of insulin-like growth factor-1-receptor (IGF1R) [21, 22].

To investigate the potential mechanisms of how metformin functions with TMZ and identify molecular changes in gene expression regulatory networks in GBM, we developed two TMZ-resistant glioblastoma cell lines, and compared proliferation, neurosphere formation, and invasion capacity of metformin treated, TMZ-resistant cells with their corresponding parental cells. Our results demonstrate that metformin might function through multiple pathways in partial restoration of TMZ sensitivity in glioblastoma cells, which subsequently enhances chemotherapy effects of TMZ.

## RESULTS

### Generation of TMZ-resistant glioblastoma cell lines

**Figure 1:**
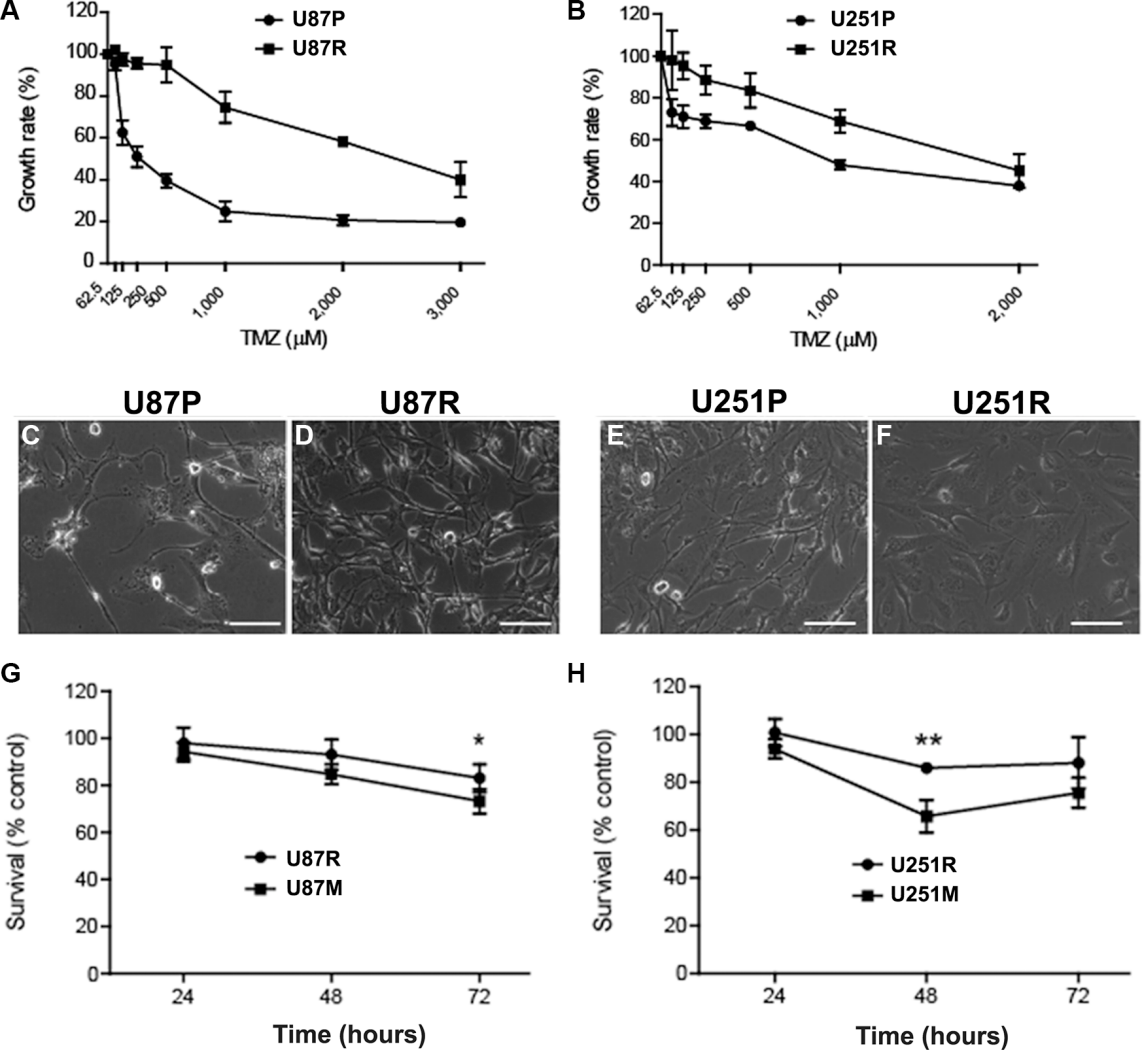
Metformin reduces temozolomide (TMZ) resistant glioblastoma cells (**A**, **B**) Generation of TMZ-resistant U87R (A) and U251R (B) glioblastoma cell lines by extended TMZ treatment. IC_50_ of resistant cells is > 2-fold higher than that of the parental lines. (**C**–**F**) As cells becomes TMZ resistant, their morphology also changes. (**G**, **H**) Metformin (1 mM) treatment is able to improve the sensitivity of both TMZ-resistant U87R (G) and U251R (H) cells as it reduces the number of surviving cells after TMZ treatment. U87M and U251M represent U87R and U251R cells that are pre-treated with metformin for 2 weeks. ^*^p = 0.072, ^**^p < 0.05. Scale bar, 100 μM.

Glioblastoma cell lines U87 and U251 (named as U87P and U251P for parental cell lines. Nomenclature of all cell lines is listed in Supplementary Table S1) were treated with TMZ with gradually increasing doses, starting from 50 μM to 600 μM, over a period of 8–10 months. IC_50_ (half or 50% minimal inhibitory concentration) was used to monitor the change of their resistance properties. Before TMZ treatment induction, IC_50_ of U87P was 325 μM. At the end of the treatment, IC_50_ has increased by 2.6 folds and reached 1,165 μM. Similarly, IC_50_ for U251P was 722 μM, while the cells obtained after TMZ induction showed an IC_50_ of 1,994 μM, a nearly 2-fold increase compared to the parental line. It is worth noting that once established, both TMZ-resistant cell lines maintained strong resistance to further TMZ treatment. These cell lines with higher IC_50_'s were therefore named U87R and U251R, respectively, and they were used in experiments described in the current work (Figure 1A, 1B). The resistant GBM cells showed similar proliferation rate and doubling time comparing to their respective parental cell lines, U87P and U251P, although changes in morphology were noted after acquisition of TMZ resistance. U87R cells showed enlarged cytoplasm and curved cellular processes. U251R cells became elongated and pleomorphic with varied sizes of the cytoplasm and bamboo-like processes (Figure 1C–1F).

### Metformin partially restores TMZ sensitivity in TMZ-resistant glioblastoma cell lines

To test whether pre-conditioning of TMZ resistant cells with metformin will be able to reverse the drug resistance, U87R and U251R cells were first treated with metformin (1 mM) for 2 weeks, then they were exposed to TMZ (50 μM) for 24, 48, and 72 h, respectively. Fifty μM of TMZ was chosen because this dosage is known to be clinically relevant [23]. Cell survival rate was measured at 24, 48, and 72 h time points. While both U87R and U251R showed a high survival rate of about 80–100% after 3 days of TMZ treatment, the survival rate dropped significantly for the cells that were pre-treated with metformin (Figure 1G, 1H). For U87R, 98%, 93.1%, and 83.1% of cells survived TMZ treatment at 24, 48, and 72 h, respectively, while the cells that were pre-conditioned with metformin (hence named as U87M) responded to TMZ treatment, and only 94.3%, 84.7%, and 73.3% of cells were viable at 24, 48, and 72 h, respectively. U251R cells exhibited a similar trend. About 100%, 86%, and 88.2% of cells survived TMZ treatment at 24, 48, and 72 h, respectively, while the cells that were pre-treated with metformin (hence named as U251M) responded to TMZ treatment, and only 94%, 65.8%, and 75.7% of cells were able to survive at 24, 48, and 72 h time points, respectively. It is interesting to note that although cell numbers kept decreasing as TMZ treatment continued over the 3-day period in U87R cells, the time course profile was different for U251R cells in that at 48 h of TMZ treatment, U251R cells showed the highest number of cell deaths (34.2% of cell deaths, and 65.8% of survival). Furthermore, TMZ treatment for 72 h or longer was not able to further reduce cell numbers, instead, allowed some cells to escape TMZ treatment. This surprising result might be due to the intrinsic differences with regards to drug response between the two cell lines. Data from longer time points is warranted to clarify this observation. Nevertheless, results from this set of experiments support the conclusion that metformin could partially restore TMZ sensitivity in TMZ-resistant glioblastoma cells. Collectively, these results suggest that pre-treatment with metformin can convert TMZ-resistant cells into a state in which they are susceptible to a clinically efficacious concentration of TMZ.

### Metformin reduces the capability of migration and invasion of TMZ-resistant glioblastoma cells

**Figure 2:**
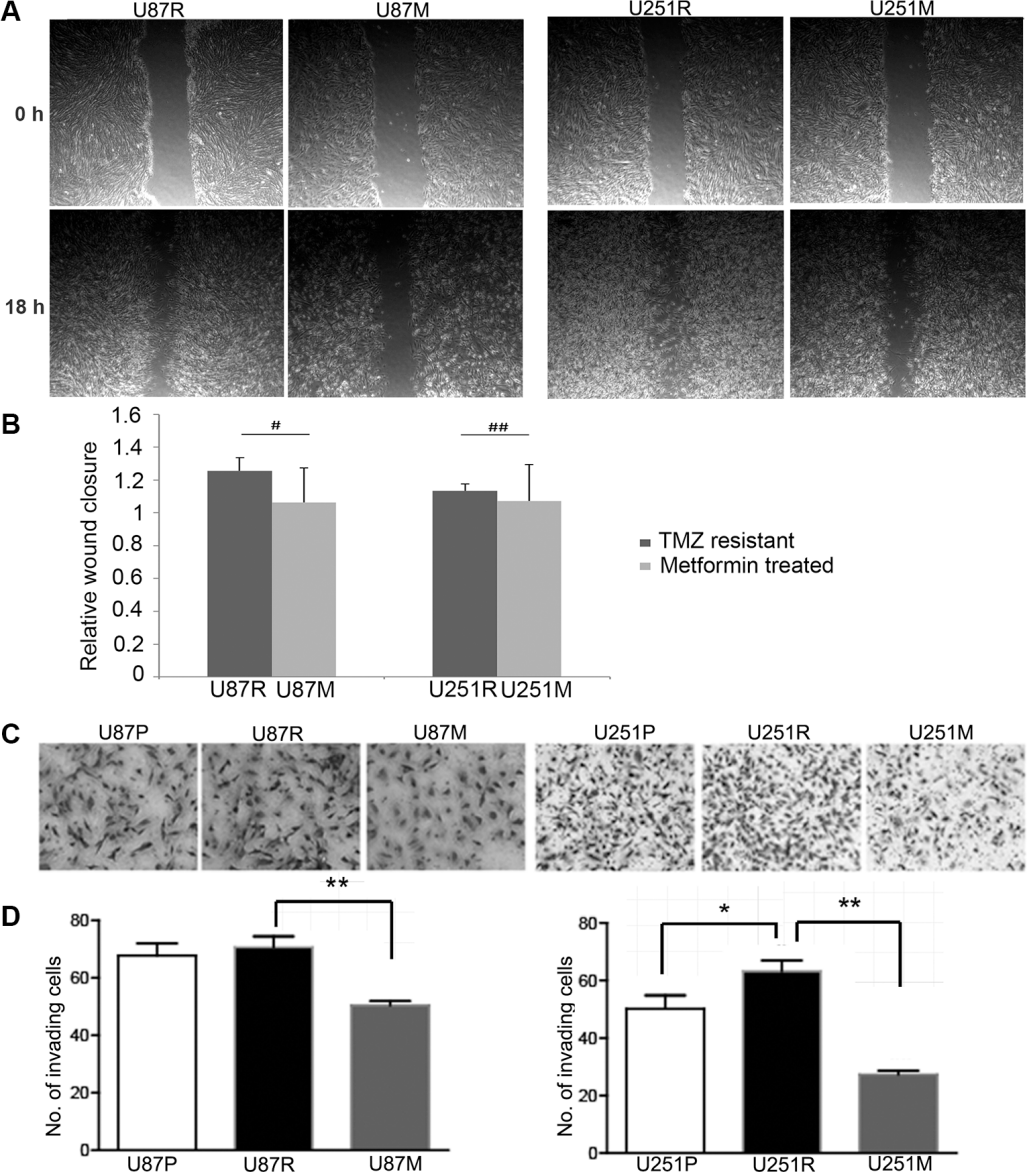
Metformin inhibits migration and growth of TMZ-resistant glioblastoma cells (**A**, **B**). Migration (scratch) assays are performed in TMZ-resistant U87R and U251R cells before and after metformin treatment (1 mM) for 2 weeks. The migration (wound healing) ability of metformin treated cells is greatly reduced at the 18 h time point for both U87R and U251R cells. (**C**, **D**) Invasion assays are performed with transwell culture in U87R and U251R cells before and after metformin treatment (1 mM) for 2 weeks. In metformin pre-treated resistant U87M and U251M cells, significantly fewer cells are able to invade through transwells as shown in phase contrast images and quantification results. ^#^*p* < 0.05, ^##^*p* = 0.07, ^*^p < 0.05, ^**^p < 0.01.

In addition to testing the effect on general response to TMZ treatment with metformin pre-conditioning, we examined the effects of metformin on both migration and invasion capabilities of TMZ resistant cells using wound-healing scratch assays. Both U87R and U251R underwent 2 weeks of metformin (1 mM) pre-treatment (hence the resultant cell lines named as U87M and U251M), and were tested for wound-healing capacity. Eighteen hours after the scratch, wound healing capacities were significantly reduced in both cell lines, reaching 10-fold (*p* < 0.05) and 3-fold (*p* = 0.07) reduction for U87M and U251M cells, respectively (Figure 2A, 2B). These results suggest that metformin was able to weaken the migration capacity of TMZ drug resistant glioblastoma cells.

To examine the effects of metformin on invasion capability of glioblastoma cells, we conducted chamber invasion assay in U87 and U251 parental (U87P and U251P), TMZ-resistant (U87R and U251R), and TMZ-resistant cells pre-conditioned with metformin (1 mM) treatment (U87M and U251M) for two weeks. Phase images of invading cells through transwell were captured and the number of invading cells was counted and analyzed (Figure 2C, 2D). We found both U87R and U251R were more capable of invading through the transwells than their parental cells. Metformin pre-treatment for two weeks, however, profoundly inhibited the capacity of tumor invasion for both U87R and U251R cells. Interestingly, the invasion capacity of metformin treated TMZ-resistant cells was even lower than the two parental lines, indicating that metformin might exert direct inhibition for cell invasion in addition to working via the pathways that are activated by TMZ.

### Metformin down-regulates SOX2 expression and reduces the formation of neurospheres in TMZ-resistant glioblastoma cells

**Figure 3:**
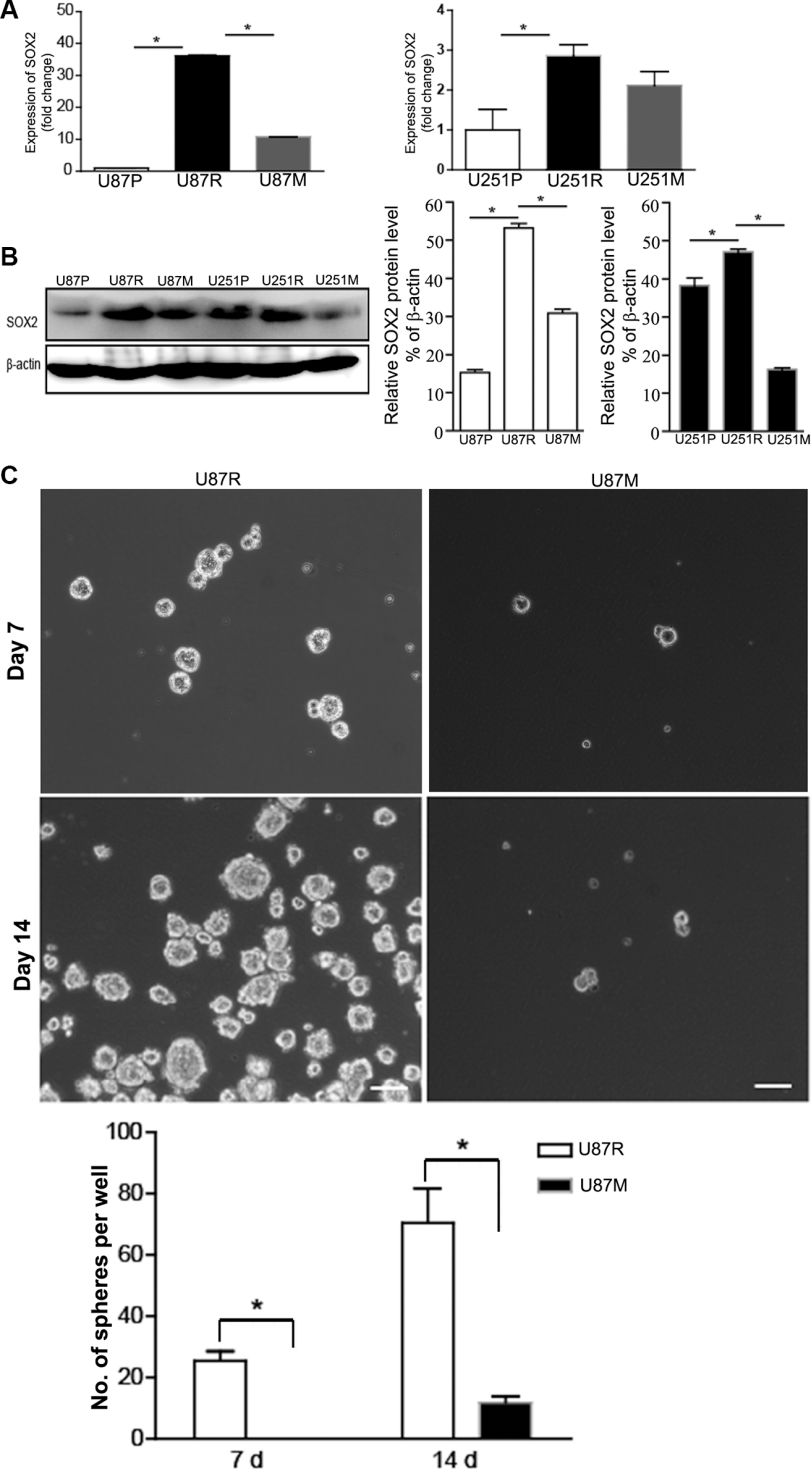
Metformin treatment partially reverses the up-regulation of SOX2 expression and inhibits neurosphere formation in TMZ-resistant glioblastoma lines (**A**) The expression level of SOX2 is measured in parental (U87P, U251P), TMZ-resistant (U87R, U251R), and metformin treated TMZ-resistant, U87 and U251 (U87M, U251M) cells by qPCR. Compared to parental lines, in TMZ-resistant lines, SOX2 expression is increased by 3 folds and 35 folds, respectively. Metformin treatment partially reduces the SOX2 expression level to near baseline. (**B**) SOX2 protein expression is measured by Western blots and the result is consistent with SOX2 mRNA level in panel A. β-actin is used as an internal control. (**C**) Neurosphere formation assays are performed on U87R and U87M cells. The number of neurospheres is counted and calculated at 7 d and 14 d of suspension culture. Phase contrast images show a drastically reduced number of neurospheres in U87M cells compared to U87R. *n* = 5 experiments. ^*^p < 0.05. Scale bar, 100 μM.

SOX2 has been reported to play a pivotal role in developing drug resistance during glioblastoma treatment [24, 25]. To test any changes of SOX2 gene expression in TMZ-resistant and metformin treated cells, we performed qRT-PCR and Western blot analysis to examine the expression of SOX2 at both the mRNA and protein levels, in U87P, U87R, and U87M, as well as U251P, U251R, and U251M, respectively. Compared to U87P and U251P, SOX2 mRNA expression was significantly greater in TMZ-resistant U87R and U251R cells. In contrast, SOX2 mRNA expression level was lower in U87M and U251M cells (Figure 3A). These findings support the hypothesis that metformin treatment partially restored TMZ sensitivity. Consistent with the qRT-PCR result, Western blot analysis showed that SOX2 protein levels were elevated in U87R and U251R, while the protein levels were reduced in U87M and U251M (Figure 3B).

SOX2 is a neural stem cell marker. Neurosphere formation assay is often used to evaluate the proliferation and self-renewal capacity of neural stem cells and glioma cancer stem cells. To test the effects of metformin on potential cancer stem cell properties, we performed neurosphere formation assays on U87P, U87R, and U87M. Since U251 cells do not form neurospheres, we only performed neurosphere formation assay on U87 cells. An equal number of cells (~100 cells/well of a 6-well plate) was grown on ultra-low attachment plates in serum free medium to encourage sphere formation. The number of neurospheres sized > 50 μm in diameter was counted at both Day 7 and Day 14 time points. TMZ-resistant cells showed robust neurosphere formation and had on average of 26 ± 4 spheres per well on Day 7 and 70 ± 10 spheres per well on Day 14 (Figure 3C). The metformin pre-treated group, U87M, however, could not form neurospheres on Day 7 and Day 14 by the same criteria for U87R. They only formed significantly reduced numbers of very tiny clumps (Figure 3C, *p* < 0.05).

### Metformin inhibits tumor growth *in vivo*

**Figure 4:**
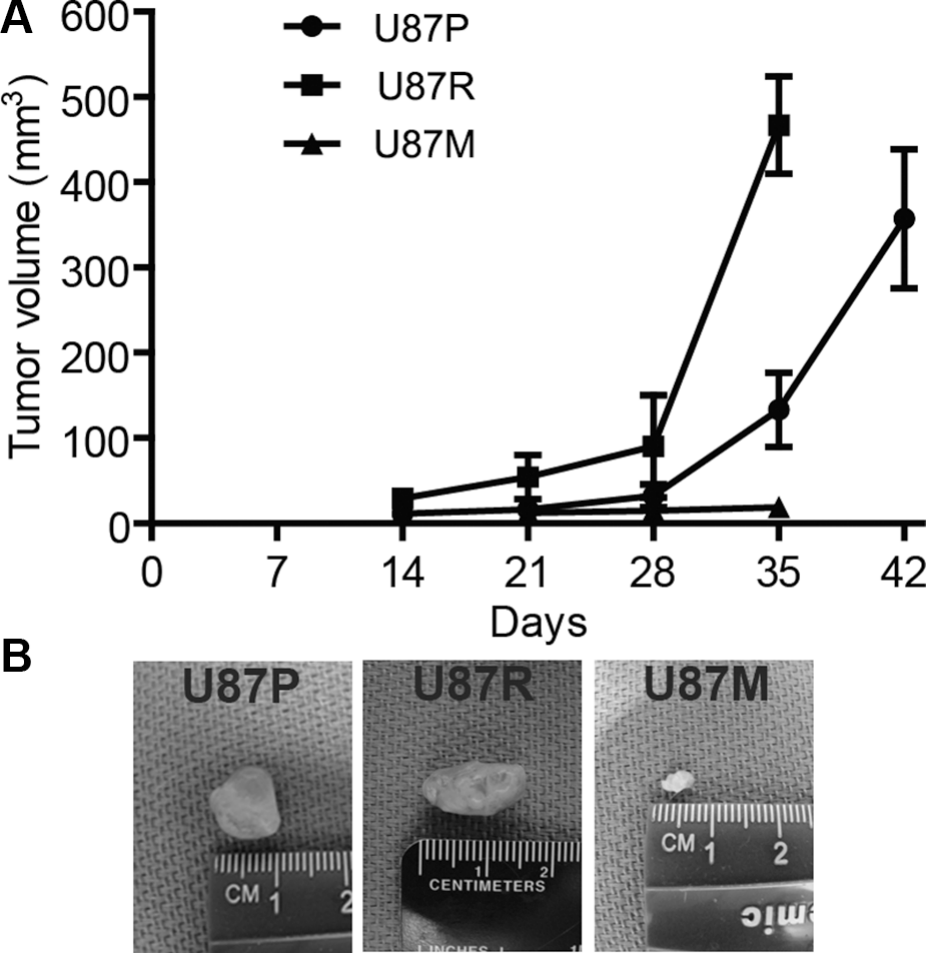
Metformin treatment reduces tumor growth in mouse xenografts (**A**) Tumor volume is measured along the time course of post injection of U87P, U87R, and U87M cells, respectively. U87P and U87R cells generate tumors of similar sizes, while U87M cells show slow growth rate and form much smaller tumors. Representative tumor images of each group are shown in (**B**).

To evaluate tumor growth capacity *in vivo*, we injected glioblastoma cells into the lower flank of severe combined immunodeficiency (SCID) mice. The mice were randomly divided into 3 groups to receive U87P, U87R, and U87M cells. Tumor volume in the U87P group gradually expanded during the first month post injection, accelerated in the second month, and reached ~360 mm^3^ at 6 weeks post injection. TMZ-resistant U87R cells generated tumors of a similar size during the first month, but in the second month, the tumor size quadrupled, indicating that TMZ-resistant cells had significantly greater growth capacity. In contrast, tumors from U87M, the metformin treated TMZ-resistant cells, showed slower growth rate and formed much smaller tumors compared to both U87P and U87R cells, as tumors generated from U87M only had a mere volume of 10 mm^3^ at 5 weeks post injection, consistent with previous reports that metformin acted to attenuate tumorigenicity of glioblastoma cells (Figure 4A, 4B).

### Global gene expression profiling reveals that multiple pathways are involved in metformin treatment related changes

To better understand the mechanism of metformin restoration of TMZ sensitivity of glioblastoma cells and identify potential pathways that contribute to TMZ resistance and the metformin reversal of such resistance in both U87 and U251 cells, we performed genome-wide gene expression profiling using Illumina bead array platform, sorted out differentially expressed genes (DEGs), and constructed networks based on DEGs. Two biological replicates of U87P, U87R, and U87M, as well as U251P, U251R, and U251M were included in the analysis.

**Figure 5:**
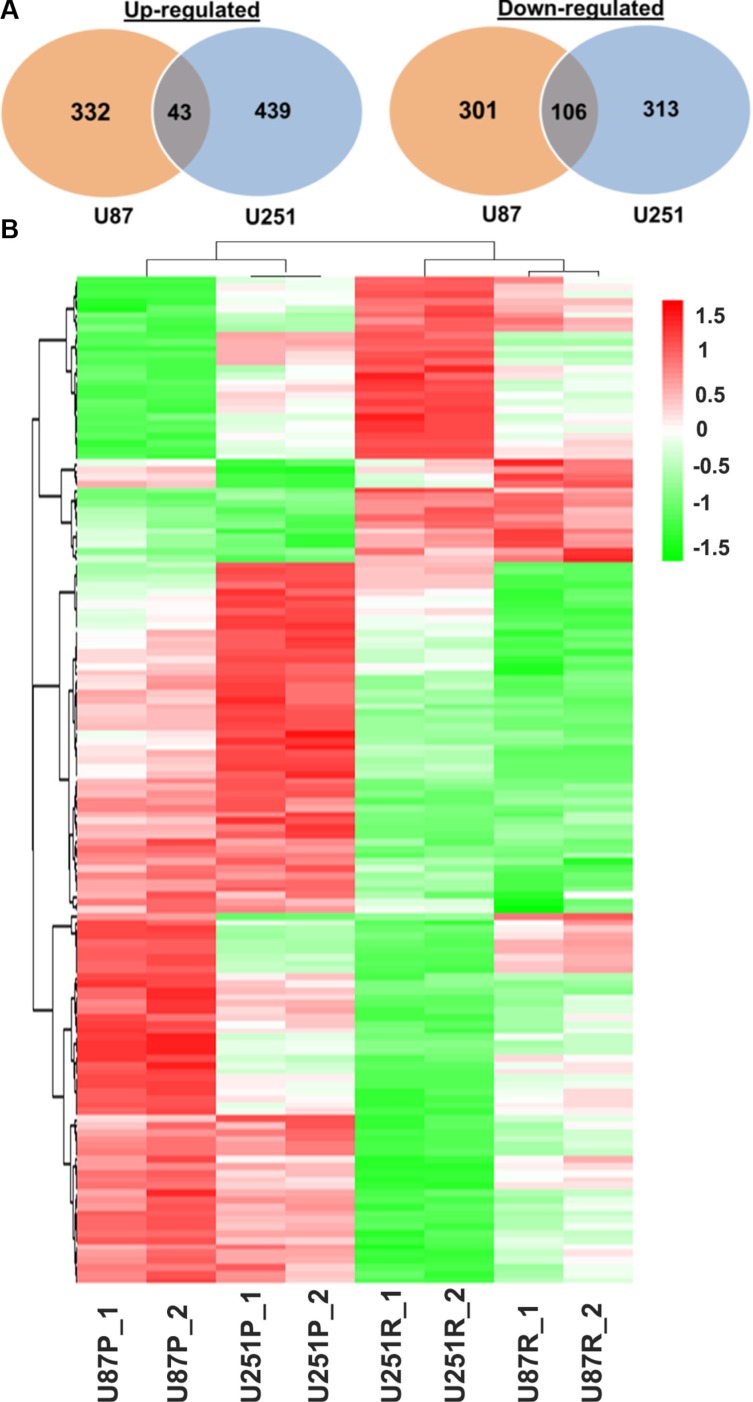
Distinct gene expression profiles of parental and TMZ-resistant glioblastoma cells (**A**) Venn diagrams show that differentially expressed genes and commonly up- or down-regulated genes in TMZ-resistant vs. parental cells U87 and U251, respectively. (**B**) Heatmap shows parental and TMZ-resistant lines cluster together. U87P_1 and U87P_2 are two biological replicates of the U87 parental line; U251P_1 and U251P_2 are two biological replicates of the U251 parental line; U87R_1 and U87R_2 are two biological replicates of the U87 TMZ-resistant line; U251R_1 and U251R_2 are two biological replicates of the U251 TMZ-resistant line.

We first obtained the gene expression profiles of TMZ-resistant and parental lines (U87R vs. U87P; U251R vs. U251P). Compared with U87P, 375 genes were up-regulated and 407 genes were down-regulated in U87R cells. In parallel, compared with U251P, 482 genes were up-regulated and 419 genes were down-regulated in U251R cells. Of these genes, 43 genes were commonly up-regulated in both U87R and U251R, while 106 genes were commonly down-regulated in both cell lines (Figure 5).

A review of the top 10 genes that were expressed higher in U87R than in U87P (Supplementary Table S2) revealed that many of the genes have been previously identified as associated with chemoresistance or have been proposed as biomarkers for a variety of tumors including gliomas and GBM, thus validating our approach of identifying these genes by microarrays. These DEGs were then analyzed for Gene Ontology (GO) terms and KEGG pathways.

Compared to U87P, in U87R lines, 4 cellular component (CC) terms involving vacuole, lysosome, and mitochondrial functions were up-regulated, demonstrating that these components might contribute to TMZ resistance. From the down-regulated DEGs list, 17 biological process (BP) terms, 6 CC terms, and 2 molecular function (MF) terms were shown. These terms are associated with wound response, inflammatory response, or cytokine activity. Most interestingly, both cell proliferation and anti-apoptosis processes were down-regulated in U87R compared to U87P, indicating the complex nature of TMZ resistance. Nucleotide-binding oligomerization domain (NOD) like receptor signaling pathway, which is involved in cell response to stress, is also down-regulated. This observation is consistent with GO analysis as several endoplasmic reticulum stress (ER stress) terms were down-regulated (Table 1).

**Table 1:** Top 5 GO terms and pathways between TMZ-resistant and parental cell lines

	Up-regulated	Down-regulated
**U87R vs. U87P**	GO:0005773~vacuole	GO:0009611~response to wounding
	GO:0005764~lysosome	GO:0006954~inflammatory response
	GO:0000323~lytic vacuole	GO:0008285~negative regulation of cell proliferation
	GO:0005739~mitochondrion	hsa04621:NOD-like receptor signaling pathway
**U251R vs. U251P**		GO:0042592~homeostatic process
		GO:0006984~ER-nuclear signaling pathway
		GO:0010941~regulation of cell death
		GO:0006916~anti-apoptosis
		GO:0051789~response to protein stimulus

In the comparison of U251R and U251P cells, no GO terms or KEGG pathways were identified in up-regulated DEGs, while 24 BP terms, 10 CC terms, 9 MF terms and 1 KEGG pathway were found to be altered from the down-regulated DEGs list. Similar to U87 lines, these GO terms were mainly involved in ER stress and anti-apoptosis (Table 1), indicating that these components are commonly involved in the development of TMZ resistance. Overlapping GO terms from the comparison of U87R vs. U87P, and U251R vs. U251P lines are all related to ER stress pathways.

**Figure 6:**
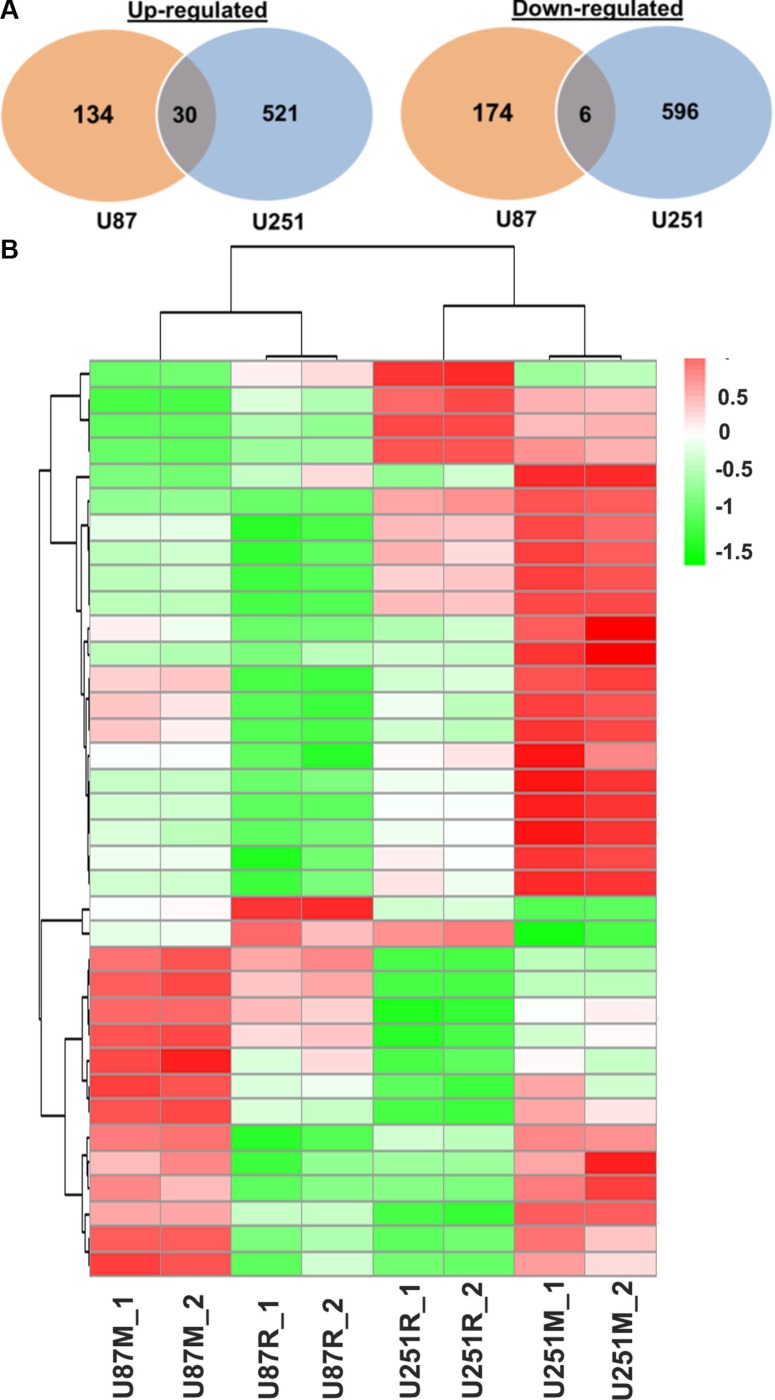
Distinct gene expression profiling of TMZ-resistant and metformin treated glioblastoma lines (**A**) Venn diagrams show that differentially expressed genes and commonly up- or down-regulated genes in metformin treated vs. TMZ-resistant cells between the two glioblastoma lines U87 and U251. (**B**) Heatmap shows TMZ-resistant lines and metformin treated lines. Metformin treated U87 and U251 show similar yet different gene expression patterns. U87M_1 and U87M_2 are two biological replicates of the U87 cells treated with metformin; U251M_1 and U251M_2 are two biological replicates of the U251 cells treated with metformin; U87R_1 and U87R_2 are two biological replicates of the U87 TMZ-resistant cell line; U251R_1 and U251R_2 are two biological replicates of the U251 TMZ-resistant cell line.

Next, we compared gene expression profiles of metformin treated and TMZ-resistant cells (U87M vs. U87R; U251M vs. U251R). Comparing U87M with U87R, 164 genes were up-regulated and 180 genes were down-regulated. In U251M compared to U251R, 551 genes were up-regulated and 602 genes were down-regulated. Among those, 30 were commonly up-regulated, and 6 genes were commonly down-regulated in both U87M and U251M cell lines (Figure 6).

The two cell lines responded to metformin treatment in a distinct manner. The top 10 DEGs are listed in Supplementary Table S3. In GO analysis of U87M vs. U87R, 5 BP terms, 4 CC terms, and 1 KEGG pathway were extracted in up-regulated DEGs, while 4 BP terms and 1 KEGG pathway were obtained from the down-regulated DEGs. The up-regulated genes were related to DNA replication and cell adhesion while the down-regulated genes were associated with steroid biosynthesis (Table 2). In GO analysis of U251M vs. U251R, 29 BP terms, 13 CC terms, and 1 KEGG pathway were obtained from up-regulated DEGs, which are mainly related to cell cycle regulations. Of the 4 BP terms, 12 CC terms, 5 MF terms, and 1 KEGG pathway extracted from the down-regulated DEGs, ribosome and protein translation pathways are the major objects of repression (Table 2).

**Table 2:** Top 5 GO terms and pathways between metformin treated and TMZ-resistant cells

	Up-regulated	Down-regulated
**U87M vs. U87R**	GO:0006260~DNA replication	GO:0016126~sterol biosynthetic process
	GO:0006259~DNA metabolic process	GO:0016125~sterol metabolic process
	GO:0006261~DNA-dependent DNA replication	GO:0006694~steroid biosynthetic process
	GO:0007155~cell adhesion	GO:0008203~cholesterol metabolic process
	GO:0022610~biological adhesion	hsa00100:Steroid biosynthesis
**U251M vs. U251R**	GO:0000278~mitotic cell cycle	GO:0006414~translational elongation
	GO:0000279~M phase	GO:0006412~translation
	GO:0022403~cell cycle phase	GO:0042254~ribosome biogenesis
	GO:0022402~cell cycle process	GO:0042274~ribosomal small subunit biogenesis
	GO:0007049~cell cycle	GO:0022626~cytosolic ribosome

### Metformin partially reverses gene expression in TMZ-resistant glioblastoma cells

**Figure 7:**
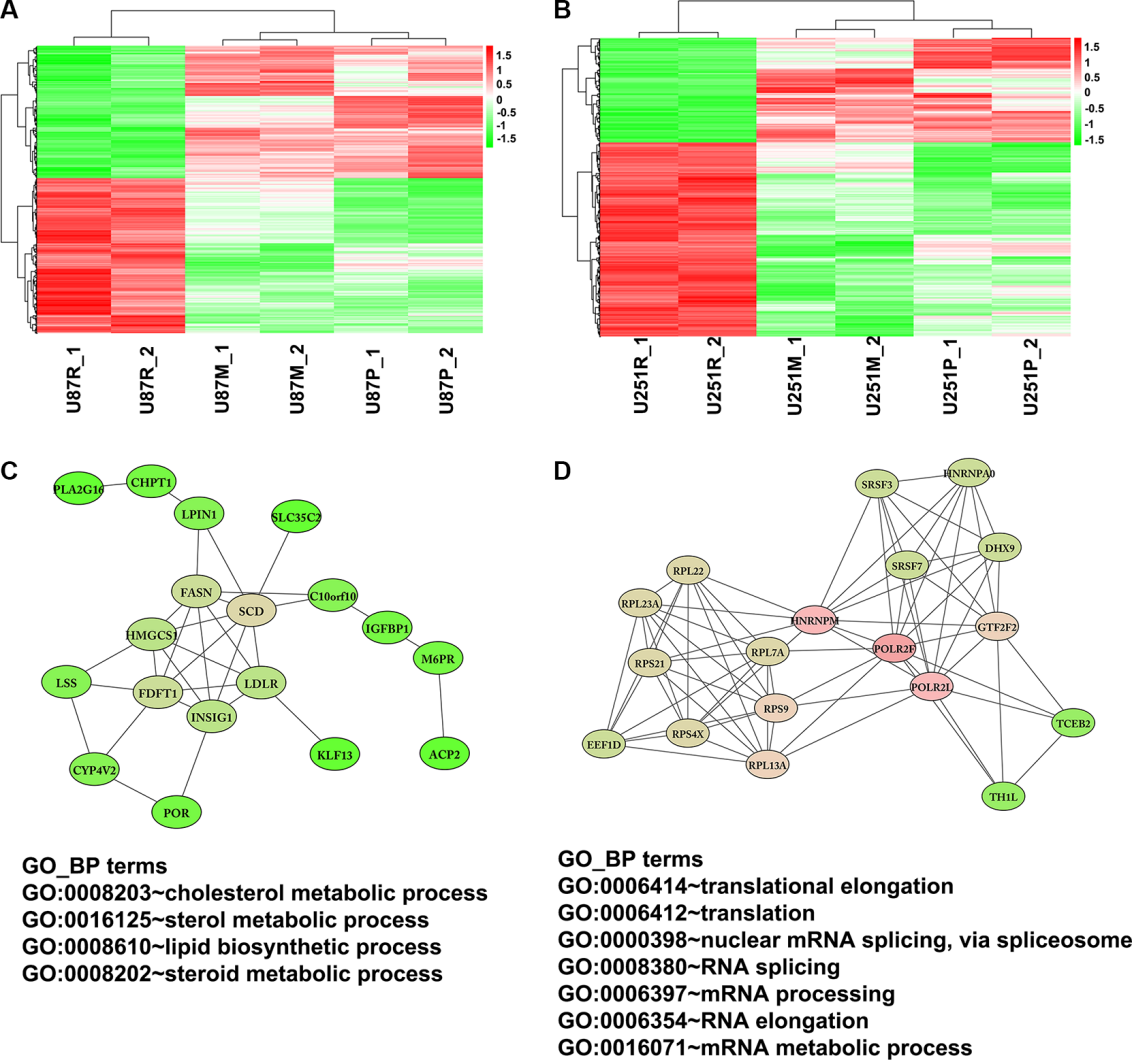
Networks extracted from co-regulated genes that are altered by TMZ treatment and reversed by metformin treatment Heatmap shows genes that are altered by TMZ treatment and reversed by metformin treatment in U87 (**A**) and U251 (**B**) cells. (**C**) A cluster of fatty acid metabolism genes was identified to be up-regulated when treated with TMZ but reversed with metformin treatment. (**D**) Another example is a network of genes that functions on translation and RNA binding and splicing. The expression of this cluster of genes was also up-regulated when treated with TMZ but reversed with metformin treatment.

To further characterize pathways on which metformin might act to reverse TMZ resistance, we identified genes that were differentially expressed (up- or down-regulated) in TMZ resistant cells which were later reversed after metformin treatment. When the cutoff was set to gene expression fold change larger than 1.2 and the *p* value was less than 0.05, a total of 415 genes were identified as being reversed by metformin treatment in U87 cell lines. Of those, 229 genes were up-regulated in U87R and reversed in U87M cells; 186 genes were down-regulated in U87R and reversed in U87M (Figure 7A, Supplementary Table S4). A total of 561 genes were identified as being reversed in U251 cell lines, with 362 genes up-regulated in U251R and reversed in U251M, and 199 genes down-regulated in U251R and reversed in U251M cells (Figure 7B, Supplementary Table S5).

To further explore potential regulatory networks among the reversed genes, we retrieved protein-protein interactions (PPI) of these genes from the String database and reconstructed the PPI network using Cytoscape software. A total of 158 reversed genes formed 367 interactions in U87 cells and 334 reversed genes formed 790 interactions in U251 cells (Supplementary Figures S1, S2). It was found these reversed genes worked together, interacting as a large network during the process of metformin reversal of TMZ resistance.

In the co-regulated genes, several networks (clusters of genes) were formed based on the analysis of MCODE, a plugin of Cytoscape that identifies highly interconnected clusters within a defined set of genes. From these tightly clustered networks, we noticed a sub-network of genes involving lipid biosynthetic and cholesterol metabolic process (Figure 7C). This sub-network comprises 18 genes, including those that are responsible for fatty acid synthesis and modifications, such as FASN (fatty acid synthase) and SCD (stearoyl-CoA desaturase or delta-9-desaturase). They are co-regulated in the same direction, i.e., up-regulated when treated with TMZ but down-regulated when treated with metformin, indicating that they play a role in drug resistance and metformin's reversal of such resistance. SCD gene, encoding Δ9 desaturase, one of the two fatty acid desaturases [26], has 8 immediate connections (edges), specifically to LPIN1 (lipin 1), low density LDLR (lipoprotein receptor), LSS (lanosterol synthase), CYP4V2 (cytochrome P450, family 4, subfamily V, polypeptide 2), POR (cytochrome P450 oxidoreductase), IGFBP1 (insulin-like growth factor binding protein 1), and INSIG1 (insulin induced gene 1).

Another closely-interconnected cluster of 18 genes was up-regulated after TMZ treatment but down-regulated after cells were treated with metformin (Figure 7D). This cluster includes 6 RNA binding and splicing proteins HNRNPM (heterogeneous nuclear ribonucleoproteins M) and HNRNPA0 (heterogeneous nuclear ribonucleoproteins A0), SRSF3 (serine/arginine-rich splicing factor 3), SRSF7 (serine/arginine-rich splicing factor 7), DHX9 (DEAH-Box Helicase 9), GTF2F2 (general transcription factor IIF subunit 2). It also has a group of translation regulatory genes including ribosomal protein RPL7A, RPL13A, RPL22, RPL23A, RPS4X, RPS9, RPS21, polymerase (RNA) II subunit POLR2L, POLR2F, and EEF1D (eukaryotic translation elongation factor 1 delta). Function enrichment analysis shows that these clustered genes participate in translational elongation and nuclear mRNA splicing.

## DISCUSSION

In this study, we generated TMZ-resistant glioblastoma cell lines and showed that metformin could partially restore TMZ sensitivity in TMZ-resistant cells. In addition, metformin reduced migration and invasion capacities of TMZ-resistant glioblastoma cells. We found that metformin down-regulated SOX2 expression in TMZ-resistant glioblastoma cells, reduced the formation of neurospheres by glioblastoma cells, and inhibited tumor growth *in vivo*. Most compelling, we performed global gene expression profiling of this set of glioblastoma cell lines, which revealed that multiple pathways might be involved in metformin treatment-related changes.

It was further shown that stem-like properties of GBM cells contribute to chemo-resistance to TMZ [27]. Transcription factor SOX2 has been identified as an oncogene in many cancers and plays important roles in cancer stem cells (CSC), EMT, and metastasis of cancer cells. The downstream mechanisms that specifically drive SOX2-dependent invasion in glioblastoma remain to be identified. Recently, it was reported that E3 ubiquitin ligase CDC20-APC interacts with and regulates SOX2 protein to promote SOX2-dependent transcription and drive glioma stem cell invasiveness and self-renewal [28]. The inhibition of cell growth was found to be due to changes in the cell cycle, and inhibition of SOX2 has led to cell cycle arrest in G2/M phase and translated into a lower cell migration rate [29]. Attenuated S-phase entry was observed in human glioma cells upon inhibition of SOX2 [30]. The mechanisms that contribute to elevated SOX2 levels in resistant cancers are not fully understood. Ectopic expression of SOX2 in breast cancer cells renders them more resistant to tamoxifen treatment *in vitro* and *in vivo*, and is associated with an increase in the frequency of stem cells and capacity for invasion [31]. Treatment of medulloblastomas in mice with an anti-mitotic drug or sonic hedgehog (SHH) pathway inhibitor resulted in residual tumors that were enriched in SOX2^+^ cells, indicating that these cells were likely to contribute toward tumor relapse [32]. SOX2 is also implicated in the cancer stem cell phenotype and development of chemo-resistance in GBM [33]. In the current work, we present that SOX2 is associated with TMZ-resistance in U87 and U251 lines, which is consistent with the important roles of SOX2 in CSC maintenance and drug resistance.

In addition, the difference in localization of the SOX2 protein as opposed to its level of expression may also be important. SOX2 has been shown to be located in the cytoplasm or both nucleus and the cytoplasm in several cancer tissues, such as lung and prostate cancer [34, 35]. However, it seemed thought that SOX2 is restricted to the nuclei of GBM cells in patient tissues or cell lines [36, 37]. Our data is consistent with published reports that SOX2 expression was restricted to the nuclei in our parental U251 cells. Further investigation along the lines of subcellular localization of SOX2 in TMZ-resistant or metformin treated cells is warranted.

Interestingly, TMZ-resistant cells treated with metformin lost their aggressiveness and showed a decrease of SOX2 expression compared with resistant cells before metformin treatment (Figure 3). Evidence demonstrating that metformin selectively targets CSCs and reverses multidrug resistance continues to accumulate. It has been shown that metformin inhibited the proliferation of CSCs and reduced neurosphere formation in GBM cells [20, 38]. When co-administered with TMZ, metformin prevented the growth of stem-like cells in gliomas [15–17]. Meanwhile, metformin also effectively targeted CSCs in several types of solid tumors, such as breast cancer, pancreatic cancer, and colorectal cancer [39–41]. The anti-proliferative effect of metformin on CSCs is mainly via activation of AMPK [42] and inhibition of AKT [20]. Other pathways or proteins such as ERK/P70S6K signaling, STAT3, and Nemo-like kinase (NLK) were also identified as targets of metformin [43–45]. Consistent with previous reports, the decreased expression of SOX2 in metformin treated U87 and U251 lines in the current study indicates that metformin reduced CSCs and TMZ resistance partially via the impairment SOX2 expression.

To further reveal the mechanisms of metformin in reversal of TMZ resistance, we performed microarray analysis on these glioblastoma cell lines. Gene expression profiling between resistance and parental cell lines showed that similar pathways were involved in the process of TMZ resistance, as ER stress and anti-apoptosis were found in both U87 and U251 cell lines. A total of 149 DEGs were identified to be involved in TMZ resistance in both cell lines. Among the 43 DEGs that were commonly up-regulated in both cell lines, many of them have been reported to be involved in chemo-resistance and tumor invasion in brain tumors. For example, MGMT and STAT3 are well known for being involved in TMZ resistance [46, 47]. Another example is CD9, a gene encoding the transmembrane protein tetraspanin, which has been reported to be up-regulated and involved in tumor cell invasion, apoptosis, and resistance to chemotherapy from transcriptomic analysis of glioblastoma tissues compared to normal brain tissues [48]. Another gene from the DEG list is Cadherin-11 (CDH11), which has been shown to promote migration of glioblastoma cells [49].

The metabolic enzyme fatty acid synthase (FASN) is also up-regulated in TMZ resistant U87 and U251 cells (U87R and U251R). FASN is a multifunctional enzyme that plays a central role in fatty acid synthesis and lipid biosynthesis [50]. In various cancers, aggressive features such as migration, invasion, metastasis, and chemo-resistance, have been shown to be dependent upon FASN [51–53]. Over-expression of FASN is also associated with glioma grade. Treatment of glioblastoma cells with FASN inhibitors resulted in a significant reduction in tumor cell viability [54]. FASN has been reported to be significantly down-regulated and decrease of FASN protein level was observed following 24 hours of metformin treatment in triple negative breast cancer cells [55]. In our current work, up-regulated FASN expression in TMZ-resistant lines was decreased after metformin treatment. Furthermore, a sub-network associated with fatty acid metabolism was also identified (Figure 7C). The expressions of 18 genes (including FASN) in this network was up-regulated in TMZ resistant cell lines and decreased after metformin treatment. In this clustered network, two proteins responsible for fatty acid synthesis and modification and encoded by FASN and SCD, acted as key regulators and interacted with other fatty acid metabolism components such as LPIN1, LDLR, IGFBP1, and INSIG1. All of these data indicate that fatty acid metabolism is involved in TMZ resistance and the potential reversal of such resistance by metformin.

Another cluster of genes reversed after metformin treatment is associated with translational elongation and RNA binding and splicing. RNA binding proteins interact with pre-mRNA when genes are actively transcribed by RNA polymerase II in ribosomes and participate in nascent mRNA splicing, nuclear export, and stability [56, 57]. Two heterogeneous ribonucleoproteins (hnRNPs) and their functionally related components are included in this subnetwork. hnRNPs are involved in multiple steps of gene transcription and subsequent posttranscriptional modification [58]. Emerging evidence has demonstrated that hnRNPs play key roles in tumor development and procession [59, 60]. We have also noticed that hnRNPM is a hub gene in the sub-network of metformin reversed genes, for it possesses over 10 edges (potential binding partners, Figure 7D). hnRNPM, an RNA splicing factor which binds GU-rich RNA cis-elements [61, 62] has been shown to promote EMT and metastasis by regulating alternative splicing in breast cancer [63]. The co-regulation of these RNA binding proteins, RNA polymerase II subunits and ribosomal proteins might contribute to the altered mRNA metabolism and ultimately TMZ resistance, while metformin treatment partially reverses this abnormal gene expression. The detailed mechanisms on how metformin regulates mRNA metabolism is warranted.

In this work, we presented differentially expressed genes and networks in glioblastoma cell lines after TMZ treatment and metformin rescue. We have realized however, that these data need to be carefully interpreted about the mechanism of action of the drug. For example, the difference in response of the cells could also be attributable to the background mutational difference. Primary GBM cells frequently contain mutations in genes such as TP53, TERT, PTEN, NF1E and EGFR. Mutations or aberrant epigenetic status of these genes in both U87 and U251 cell lines warrant further investigation. In addition, we have noticed that expression level of certain neural lineage transcription factors varied in the two glioma cell lines tested. For example, SOX2 expression was quite low in untreated U87P yet more than 2-fold higher in U251P. Such variance in critical neural transcription factors could contribute to the difference in response to metformin treatment.

In summary, we have used combinatorial experimental and bioinformatics approaches to identify gene regulatory networks that are involved in the development of TMZ resistance and the restoration of sensitivity by metformin treatment. SOX2 up-regulation, altered fatty acid metabolism, and mRNA metabolism might contribute to TMZ resistance, while metformin treatment partially reverses these up-regulated genes back to original or near original levels found in parental glioblastoma cell lines. Our findings provide potential insight into the TMZ and metformin treatment mechanism in GBM, which could in turn yield possible translational value for clinical applications. Finally, our work offers an experimentally tractable model that may facilitate personalized treatment for GBM patients and in so doing also help identify novel and/or personalized therapeutic targets, leading to potentially more effective treatment.

## MATERIALS AND METHODS

### Cell culture and generation of TMZ-resistant cell lines

Human glioblastoma cell lines U-87MG originally obtained from American Type Culture Collection (ATCC) and U-251MG originally obtained from Sigma-Aldrich were cultured in EMEM supplemented with 10% fetal bovine serum, 1% MEM NEAA (Life Technologies) and 1% GlutaMAX (Life Technologies) at 37°C and 5% CO2 in a humidified incubator. TMZ (Sigma-Aldrich) was dissolved in dimethyl sulfoxide (DMSO) to prepare a stock concentration of 200 mM which was further diluted in cell culture medium to working concentrations. To generate TMZ-resistant cell lines, U87 and U251 parental cells were initially cultured in a medium containing a clinically equivalent concentration of TMZ (50μM). The dosage of TMZ was slowly escalated by 2 times every 1 or 2 passages and reached up to 600 μM. Cells in sister dishes not treated with TMZ were cultured in parallel as controls. To investigate the effects of metformin on TMZ-resistant cells, both TMZ-resistant U87 and U251 cells were treated with metformin (1 mM) for 2 weeks.

### Neurosphere formation assay

Cells were cultured in suspension at a density of 100 cells/well in 6-well plates. Cells were cultured for 14 days in a neural stem cell medium, consisting of Neurobasal (Life Technologies) and DMEM/F12 media (HyClone) (1:1), supplemented with 1x B27, 1xN2, basic fibroblast growth factor (bFGF, 20 ng/ml), and epidermal growth factor (EGF, 20 ng/ml). The number of neurospheres/well was determined by counting five different wells on Day 7 and Day 14. Neurospheres with diameters > 50 μm were counted.

### MTT assay

The proliferation potential of cells was measured using MTT assay kit (Life Technologies). The absorbance at a wavelength of 540 nm was measured with a microplate reader.

### Wound-healing assay

The wound was created by scraping cultured glioblastoma cells with a 200 μl pipette tip. The healing process was monitored at 0 and 18 h under a microscope. Wound closure was evaluated using WimScratch Wimasis Image Analysis (www.wimasis.com).

### Invasion assay

*In vitro* invasion assays were performed in a 24-well Multiwell Insert System (BD Falcon) which contains 8 μm pore size membranes. About 100,000 cells were seeded to the top of the upper wells (previously coated with Matrigel, 1:100). The lower chamber was filled with culture medium. After 72 h of incubation, cells that migrated to the lower side of the membrane were fixed, stained with Giemsa, and counted. Each experiment was performed in triplicate.

### Quantitative RT-PCR (qRT-PCR)

qRT-PCR analysis was used to determine the mRNA expression level of SOX2 in parental, TMZ-resistant, and metformin treated glioblastoma cells. Total RNA was extracted from cells using RNeasy Mini Kit (Zymo Research). cDNA synthesis was accomplished using SuperScript III First-Strand Synthesis System (Life Technologies) following the manufacturer's instructions. Quantitative PCR was performed by using iQ™ SYBR® Green Supermix (Bio-Rad) in 7900HT Fast Real-Time PCR System (Applied Biosystems). Gene expression levels were compared after normalization to endogenous GAPDH. The primer sequences used in this study are illustrated in Supplementary Table S6. Experiments were performed in triplicate, and the results were calculated with the 2^−ΔΔCt^ method.

### Western blotting

Total protein was extracted using a PhosSTOP EASYpack (Roche) according to manufacturer's instructions. The proteins were separated by SDS-PAGE, transferred to nitrocellulose membranes, and detected with antibodies against SOX2 (Cell Signaling) and β-actin (Sigma). Immunoreactivity was detected using the ECL chemiluminescence system and quantified using an imaging densitometer. The density of each band was quantified using Quantity One software (Bio-Rad).

### Microarray experiment and gene expression analysis

Parental cells, TMZ-resistant cells, and metformin treated cells of U87 and U251 were grown in adherent conditions. RNA was extracted with the RNeasy Mini Kit (Zymo Research). RNA concentration and quality were determined with an Agilent Bioanalyzer. RNA was labelled using the Illumina TotalPrep RNA Amplification Kit (Ambion). Microarray data were converted into recognizable format and annotated with software GenomeStudio. The probes detected with *p*-value lower than 0.01 in at least one sample were accepted as significant and used for further analysis. After variance-stabilizing transformation (VST) and normalization with Robust Spline Normalization (RSN) method with package lumi of R, differential analysis were performed using package limma, respectively [64, 65]. Heatmap and clustering were generated using package pheatmap. Fold change > 1.5 and adj. *P* < 0.05 were set as the cut-offs to screen out differentially expressed genes (DEGs). The functional enrichment analysis of DEGs was performed by DAVID (The Database for Annotation, Visualization and Integrated Discovery) to identify GO categories in biological process and KEGG signal [66]. The FDR < 0.05 was used as the cut-off. The protein-protein interaction was retrieved from STRING database and reconstructed in Cytoscape software [67, 68]. Separate networks with fewer than 10 nodes were not included for further analysis. Sub-clusters analysis was performed by MCODE plugin [69].

### Xenograft mouse models

U87 parental cells, TMZ-resistant cells, or TMZ-resistant cells treated with metformin (1 mM) for 2 weeks (2 × 10^6^ cells per transplantation) were transplanted subcutaneously to the lower flank of 6~8 week-old SCID mice. Tumor growth was monitored twice weekly for up to 6 weeks. Tumor diameter was measured with calipers, and the tumor volume was calculated (length × width × width × 0.5). The survival time of the mice was recorded and the median survivals were calculated. All animal procedures were approved by The University of Texas Health Science Center at Houston (UTHealth) Animal Welfare Committee.

### Statistics

Results were compared between the groups using unpaired *t*-test. Overall survival was analyzed using the Kaplan-Meier method. Statistical analysis was performed with the SPSS software package.

## SUPPLEMENTARY MATERIALS






