# Supplementary material for: Metformin treatment reduces temozolomide resistance of glioblastoma cells
Source: Oncotarget. 2016 Oct 24;7(48):78787–803. doi: 10.18632/oncotarget.12859 (PMC5346677; doi:10.18632/oncotarget.12859)
Supplement: Supplementary file 1 [file oncotarget-07-78787-s001.pdf]

Metformin treatment reduces temozolomide resistance of glioblastoma cells

Supplementary Materials

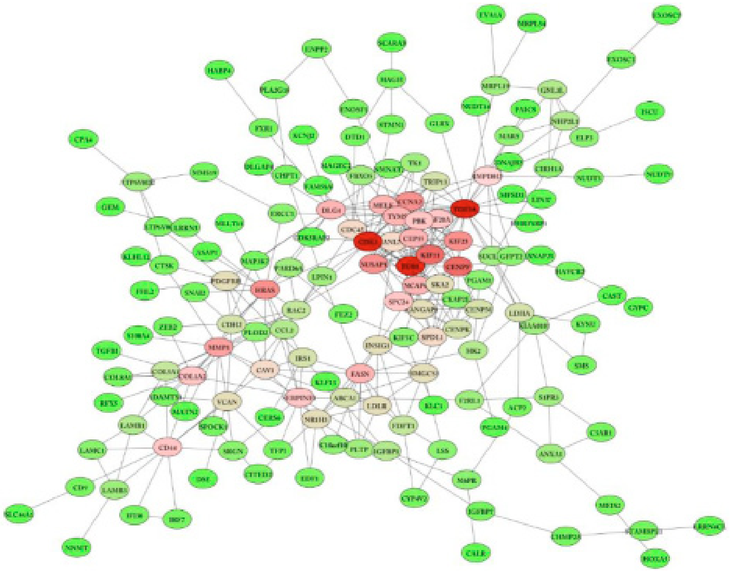

Supplementary Figure S1: Protein-protein interaction (PPI) network generated by genes whose expression was restored toward the expression levels in parental lines in U87 cells. Red nodes indicate a protein has more interactions with other proteins in the network, while green nodes indicate a protein has fewer interactions with other proteins in the network.

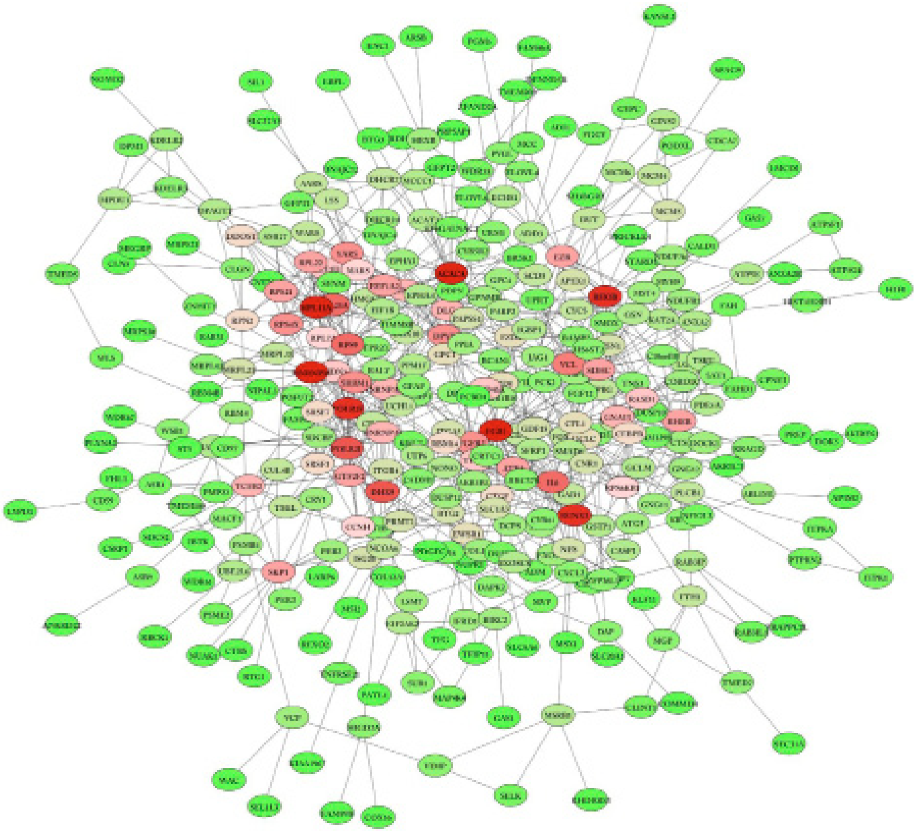

Supplementary Figure S2: Protein-protein interaction (PPI) network generated by genes whose expression was restored toward the expression levels in parental lines in U251 cells. Red nodes indicate a protein has more interactions with other proteins in the network, while green nodes indicate a protein has fewer interactions with other proteins in the network.

Supplementary Table S1: Nomenclature of cell lines and samples for Illumina bead array

No.	Line name	Sample name	Description
1	U87P	U87_P_1	Parental U87
2	U87P	U87_P_2	Parental U87 replicate
3	U87R	U87_R_1	TMZ-resistant U87
4	U87R	U87_R_2	TMZ-resistant U87 replicate
5	U87M	U87_M_1	Metformin treated TMZ-resistant U87
6	U87M	U87_M_2	Metformin treated TMZ-resistant U87 replicate
7	U251P	U251_P_1	Parental U251
8	U251P	U251_P_2	Parental U251 replicate
9	U251R	U251_R_1	TMZ-resistant U251
10	U251R	U251_R_2	TMZ-resistant U251 replicate
11	U251M	U251_M_1	Metformin treated TMZ-resistant U251
12	U251M	U251_M_2	Metformin treated TMZ-resistant U251 replicate

Supplementary Table S2: Top 10 differentially expressed genes between TMZ-resistant and parental cell lines

	Up-regulated				Down-regulated			
	**Symbol**	**logFC**	**Fold increased**	**adj.p.val**	**Symbol**	**logFC**	**Fold decreased**	**adj.p.val**
U87R vs. U87P	CHI3L1	2.87	7.31	2.21E-06	IL8	−4.38	20.82	3.05E-07
	HRK	1.84	3.59	4.57E-06	CXCL2	−3.23	9.36	1.10E-08
	MAFB	1.84	3.57	6.88E-08	IL1F8	−2.81	7.04	1.97E-08
	CT45A4	1.69	3.24	4.26E-07	HSPA5	−2.79	6.90	9.93E-08
	MAGEC2	1.66	3.15	2.95E-08	IL1B	−2.76	6.79	2.59E-07
	SPRR2D	1.53	2.89	8.03E-07	IL6	−2.60	6.06	1.55E-06
	CDH11	1.51	2.84	3.75E-06	SLC25A24	−2.58	5.98	4.11E-09
	SPINK1	1.49	2.82	4.69E-06	NFKBIZ	−2.31	4.96	2.95E-08
	VCX3A	1.48	2.80	6.05E-06	FGF2	−2.25	4.75	1.53E-05
	VCX	1.47	2.77	1.69E-05	LOC387763	−2.21	4.63	2.59E-07
U251R vs. U251P	PRSS35	3.89	14.81	2.00E-08	BEX2	−3.28	9.72	2.00E-08
	S100A4	2.99	7.92	2.34E-08	HMOX1	−3.06	8.34	1.68E-07
	LDLR	2.59	6.00	2.96E-07	TRIB3	−2.80	6.98	2.38E-06
	GFAP	2.47	5.55	1.28E-06	WARS	−2.78	6.88	9.74E-08
	CHN1	2.41	5.31	5.91E-08	CBS	−2.71	6.55	1.26E-07
	TXNIP	2.25	4.74	1.00E-05	LOC400750	−2.56	5.89	5.91E-08
	PCDH20	2.18	4.54	2.10E-07	ASNS	−2.53	5.78	3.82E-06
	SULF1	2.15	4.45	3.85E-05	HSPA5	−2.39	5.23	1.40E-07
	SOX2OT	2.12	4.35	7.02E-07	DDIT3	−2.36	5.15	1.30E-06
	FABP7	2.11	4.32	7.57E-08	HYOU1	−2.30	4.92	8.37E-07

Notes: logFC: log fold change; adj.p.val: adjusted *p* value.

Supplementary Table S3: Top 10 genes between metformin treated and TMZ-resistant cell lines

	Up-regulated				Down-regulated			
	Symbol	logFC	Fold increased	adj.p.val	Symbol	logFC	Fold decreased	adj.p.val
U87M vs. U87R	ADA	1.63	3.10	1.07E-06	SPRR2D	−3.94	15.38	1.49E-13
	DCBLD2	1.53	2.89	1.88E-03	SPRR2F	−2.61	6.11	1.37E-10
	SBSN	1.42	2.67	5.29E-05	IL6	−1.82	3.53	9.55E-08
	EFEMP1	1.39	2.63	1.93E-04	BEX2	−1.66	3.16	1.08E-09
	NDP	1.35	2.54	8.79E-06	LDLR	−1.59	3.01	5.64E-08
	MCM4	1.19	2.28	1.75E-05	GDF15	−1.56	2.94	8.64E-07
	TNFRSF21	1.14	2.21	1.79E-04	SH3BGR	−1.54	2.90	5.59E-09
	COL5A2	1.09	2.13	9.64E-05	P8	−1.52	2.86	1.41E-08
	CPA4	1.07	2.10	1.17E-04	SCD	−1.51	2.85	4.30E-09
	GJA1	1.05	2.07	7.55E-05	INSIG1	−1.50	2.84	6.11E-08
U251M vs. U251R	IFI27	6.02	65.08	2.34E-10	LOC100 132673	−2.72	6.60	2.00E-08
	IFITM1	4.81	28.07	3.11E-10	IFI6	−2.62	6.16	8.79E-07
	MX1	4.41	21.29	5.81E-10	MIR1974	−2.36	5.13	3.86E-07
	IFI6	4.40	21.07	1.02E-09	LOC91561	−2.25	4.76	8.97E-06
	ISG15	4.28	19.46	8.33E-09	LOC729679	−2.23	4.70	3.64E-07
	IFI44L	4.26	19.20	6.56E-10	LOC441506	−2.15	4.45	1.50E-06
	TXNIP	4.20	18.35	2.15E-07	LOC387867	−2.13	4.38	2.95E-07
	LOC100 129681	4.20	18.33	1.38E-09	LOC648249	−2.11	4.30	3.79E-07
	IFIT1	4.19	18.28	1.50E-08	LOC388707	−2.00	3.99	5.84E-07
	LY6E	4.18	18.12	2.26E-07		−1.97	3.92	6.90E-08

Supplementary Table S4: Genes that are altered in U87R and reversed in U87M

Supplementary Table S5: Genes that are altered in U251R and reversed in U251M

Supplementary Table S6: The primer sequences used in this study

Gene	Forward primer (5′-3′)	Reverse primer (5′-3′)
**SOX2**	CACACTGCCCCTCTCAC	TCCATGCTGTTTCTTACTCTCC
**GAPDH**	CTGGTAAAGTGGATATTGTTGCCAT	TGGAATCATATTGGAACATGTAAACC
